# Editorial of Special Issue “Protective and Detrimental Role of Heme Oxygenase-1”

**DOI:** 10.3390/ijms20194744

**Published:** 2019-09-24

**Authors:** Valeria Sorrenti

**Affiliations:** Department of Drug Science, Biochemistry Section, University of Catania, 95125 Catania, Italy; sorrenti@unict.it; Tel.:+39-0957-3741-15

The Special Issue, “Protective and Detrimental Role of Heme Oxygenase-1”, of the *International Journal of Molecular Sciences*, includes original research papers and reviews, some of which were aimed to understanding the dual role (protective and detrimental) of HO-1 and the signaling pathway involved. Heme oxygenase (HO)-1 is known to metabolize heme into biliverdin/bilirubin, carbon monoxide, and ferrous iron, and it has been suggested to demonstrate cytoprotective effects against various stress-related conditions. HO-1 is commonly regarded as a survival molecule, exerting an important role in cancer progression and its inhibition is considered beneficial in a number of cancers. However, increasing studies have shown a dark side of HO-1, in which HO-1 acts as a critical mediator in ferroptosis induction and plays a causative factor for the progression of several diseases [1]. Lackani et al. demonstrated for the first time that HO-1 has the ability to restore cellular redox, rescue SIRT1, and prevent Ang II-induced impaired effects on adipocytes and the systemic metabolic profile [2]. The study of Fujiwara et al. demonstrated that the physiological effects of the HO-1/CO system were employed for preserving donor lungs with unique characteristics via the high-pressure gas (HPG) preservation method. This approach has significant potential to be used as a new preservation method for lungs [3]. The pharmacological activation of HO-1 activity mimics the effect of caloric restriction (CR), while the HO-1 inhibitor Tin-mesoporphyrin IX (SnMP) increased oxidative stress and cardiac hypertrophy. These data suggest the critical role of HO-1 in protecting the diabetic heart [4]. Bilirubin (BR), the end product of the heme degradation pathway is an important endogenous antioxidant, and it plays a crucial role in protection against oxidative stress. OH-1 activity can modulate BR levels. Decreased inflammatory status has been reported in subjects with mild unconjugated hyperbilirubinemia. Valaskova et al. reported that hyperbilirubinemia in Gunn rats is associated with an attenuated systemic inflammatory response and decreased liver damage upon exposure to Lipopolysaccharide (LPS) [5]. Antigen-presenting cells (APCs) including dendritic cells (DCs) play a critical role in the development of autoimmune diseases by presenting self-antigen to T-cells. It has been reported that the protective effect and the reduction of lesions in the pancreas were due to the inhibition of oxidative stress mediated by HO-1 activity. Data obtained by Pogu et al. demonstrated the potential of induction of HO-1 expression in DCs as a preventive treatment, and potential as a curative approach for Type I diabetes [6]. Given the association between inflammation and prostate cancer (PCa), and the anti-inflammatory role of heme oxygenase 1 (HO-1), the study of Leonardi et al. identified an interaction between HO-1 and glucocorticoid receptor (GR). The modulation between HO-1 and GR pathways may represent a therapeutic strategy in PCa therapy [7]. Gall et al. review the heme–heme oxygenase–endoplasmic reticulum (ER) stress relationship; the major mechanisms of their interactions by which ER stress contributes to the cell and organ damage in diabetes, atherosclerosis, and brain hemorrhage. Since HO-1 presents a unique Janus-faced character in brain pathologies, this issue has received special attention [8]. The review by Kishimoto et al. summarizes the roles of HO-1 in atherosclerosis and focuses on the clinical studies that examined the relationships between HO-1 levels and atherosclerotic diseases [9].

Other original research papers of the Special issue were aimed at the identification of natural molecules or new synthetic compounds able to modulate HO-1 activity/expression. These articles will help make HO-1 a potential therapeutic target for the amelioration of various diseases. It has been reported that hepatoprotective effect of *Myristica fragrans* kernels in the livers of rats exposed to Acetaminophen (APAP)-induced hepatotoxicity could be linked to their ability to promote the NF-E2-related factor 2 (Nrf2)/ antioxidant responsive element (ARE) pathway. Hepatoprotection effects were mediated via suppressing oxidative stress, inflammation, and apoptosis [10]. A lot of evidence showed that HO-1 induces ferroptosis through an increase of ROS production mediated by iron accumulation and accompanied by augmentation lipid peroxidation and glutathione depletion. Results obtained in the study of Acquaviva et al. demonstrated that, highest concentration of *Betula etnensis Raf. (Birch Etna)* extract, was able to induce ferroptotic cancer cell death. HO-1 mediated ferroptosis may represent a chemotherapeutic strategy against tumor [11]. Metformin (MET), a drug widely used for type 2 diabetes, has recently gained interest for treating several cancers. Disrupting antioxidant HO-1 activity, especially under low glucose concentrations, could be an attractive approach to potentiate metformin antineoplastic effects, and could provide a biochemical basis for developing HO-1-targeting drugs against solid tumors [12]. Data obtained by Sorrenti et al., demonstrated that inducible nitric oxide synthase/gamma-Glutamyl-cysteine ligase (iNOS/GGCL) and dimethylarginine dimethylaminohydrolase (DDAH) dysregulation may play a key role in high glucose mediated oxidative stress, whereas HO-1 inducers such as Caffeic acid phenethyl ester (CAPE) or its more potent derivatives may be useful in diabetes and other stress-induced pathological conditions [13]. The study of Moreno et al. reveals an interaction between HO-1 and nitric oxide synthase-1 (NOS1)/ nitric oxide synthase-2 (NOS2) during peripheral inflammation and shows that Cobalt protoporphyrin (CoPP) and CO-releasing molecules-2 (CORM-2) improved HO-1 expression and modulated the inflammatory and/or plasticity changes caused by peripheral inflammation in the *locus coeruleus* [14].

Overall, the 14 contributions published in this Special Issue highlight the dual role (protective and detrimental) of HO-1 and the signaling pathways involved. HO-1 may represent a potential therapeutic target for the amelioration of various diseases. Natural molecules or new synthetic compounds able to modulate HO-1 activity/expression may represent a therapeutic strategy against various diseases.
